# Establishing an MRI-Based Protocol and Atlas of the Bearded Dragon (*Pogona vitticeps*) Brain

**DOI:** 10.3389/fvets.2022.886333

**Published:** 2022-05-13

**Authors:** Kari D. Foss, Krista A. Keller, Spencer P. Kehoe, Bradley P. Sutton

**Affiliations:** ^1^Department of Veterinary Clinical Medicine, College of Veterinary Medicine, University of Illinois Urbana-Champaign, Urbana, IL, United States; ^2^Wildlife Epidemiology Laboratory, College of Veterinary Medicine, University of Illinois Urbana-Champaign, Urbana, IL, United States; ^3^Department of Bioengineering, Beckman Institute for Advanced Science and Technology, Grainger College of Engineering, University of Illinois Urbana-Champaign, Urbana, IL, United States

**Keywords:** bearded dragon, MRI, brain, neurology, exotic, atlas

## Abstract

The bearded dragon (*Pogona vitticeps*) has become a popular companion lizard, and as such, clients have increasingly come to expect the application of advanced diagnostic and therapeutic options in their care. The purpose of this study was to establish an MRI-based protocol and brain atlas to improve diagnostic capabilities in bearded dragons presenting with neurologic dysfunction. Using a high-field 3T magnet, *in vivo* MRI of the brain was successfully performed in seven healthy bearded dragons utilizing an injectable anesthetic protocol utilizing intravenous alfaxalone. From this, we created an atlas of the brain in three planes, identifying nine regions of interest. A total scan time of 35 min allowed for the collection of a quality diagnostic scan and all lizards recovered without complication. This study provides practitioners a neuroanatomic reference when performing brain MRI on the bearded dragon along with a concise and rapid MRI protocol.

## Introduction

Advanced imaging methods, such as computed tomography (CT) and magnetic resonance imaging (MRI) are increasingly performed in the diagnosis of exotic animals. They provide a means of improving diagnostic accuracy, assisting with determining prognosis, and selecting optimal treatments. While these methods have been routinely applied to companion small animals, they require modifications in regard to exotic species due to their differing anatomy. Additionally, their small body weight may also influence the quality of MRI and CT (1).

As the ownership of exotic pets, such as the beard dragon increases, they are evaluated more frequently by veterinarians and clients are more willing to pursue advanced diagnostics, such as CT or MRI. Computed tomography (CT) is more frequently selected due to its wider availability, lower cost, shorter scan time, and ability to utilize sedation for restraint. Additionally, CT provides superior spatial resolution and is better suited for imaging bone (2). Magnetic Resonance Imaging (MRI) provides superior contrast resolution and is better suited for imaging of soft tissue structures. Images can also be acquired in multiple planes, compared to CT which are typically acquired in a single plane (2). Unfortunately, MRI has a longer image acquisition time an often requires general anesthesia. While general anesthesia poses a risk in all animals, it is greater in many exotic species and has been specifically evaluated in rabbits (1, 3, 4). At this time there are no reports assessing the risks of mortality surrounding reptile anesthesia however this taxon presents unique anesthetic challenges including intracardiac shunting, poikilothermic nature, and often challenging venous access (5–7).

While there are previously established reptilian brain MRI atlases including the tokay gecko (*Gekko gecko*), the green anole (*Anolis carolinensis*), the garter snake (*Thamnophis sirtalis*), and the tawny dragon (*Ctenophorus decressii*) many of them have been performed either on cadavers and none exist on the bearded dragon (8–12). Therefore, the development of a specific MRI protocol and brain atlas, as well as a safe anesthetic protocol are needed to aid in the diagnostic workup up of bearded dragons presenting with neurologic disease. The purpose of this study is to establish an MRI-based protocol and brain atlas to improve diagnostic capabilities in bearded dragons presenting with neurologic dysfunction.

## Materials and Methods

Before the study, test scans were performed on two bearded dragon cadavers euthanized for non-neurologic disease in order to optimize the protocol. All live animal use and experimental procedures were approved by the University of Illinois at Urbana-Champaign Institutional Animal Care and Use Committee.

### Animals

Seven captive-reared bearded dragons (four males and three females) currently part of a research and teaching colony were utilized. All animals were between 3 and 4 years of age at the time of the study and were healthy based on daily observations for the prior 3 years and the lack of detectable abnormalities on a physical examination. Mean ± SD body weight was 3,412 ± 52 g (359 ± 11 g for males and 319 ± 82 g for females). The reptiles were housed at a campus laboratory animal services facility under husbandry standard for the species (13). Each bearded dragon was individually housed in a front opening polyethylene commercial enclosure (Vision V332 Cage) measuring 91.45 × 71.12 × 45.72 cm located within a temperature controlled room (24–25.5°C). Each enclosure has a dedicated heat lamp and a dedicated ultraviolet emitting bulb (both UVA and UVB) situated above each habitat and 12 h of darkness were provided daily with a basking temperature reaching 35–37°C. Daily diet offered included a variety of dark leafy greens (collard greens, kale, turnip greens, mustard greens) with additional shredded produce (carrots, sweet potato, berries) supplemented with calcium carbonate powder and twice weekly offering of gut loaded king worm (*Zophobas morio*) larvae or dubia roaches (*Blaptica dubia*). Food was withheld for 24 h prior to the imaging study.

### Magnetic Resonance Imaging Protocol

All reptiles were transported to the Beckman Institute Biomedical Imaging Center in individual covered containers with holes for ventilation for which the lizards were previously acclimated. Upon arrival, the reptiles remained in their separate containers on external heat sources to maintain an ambient temperature of ~27°C. Prior to MRI acquisition, the reptiles were anesthetized with 15 mg/kg alfaxalone (Alfaxan^®^, Jurox Inc., Kansas City, MO) IV in the coccygeal vein administered over 20–30 s. This protocol has been shown to deliver a light plane of anesthesia for 30–45 min without clinically significant cardiovascular or respiratory effects (14).

**Figure 1:**
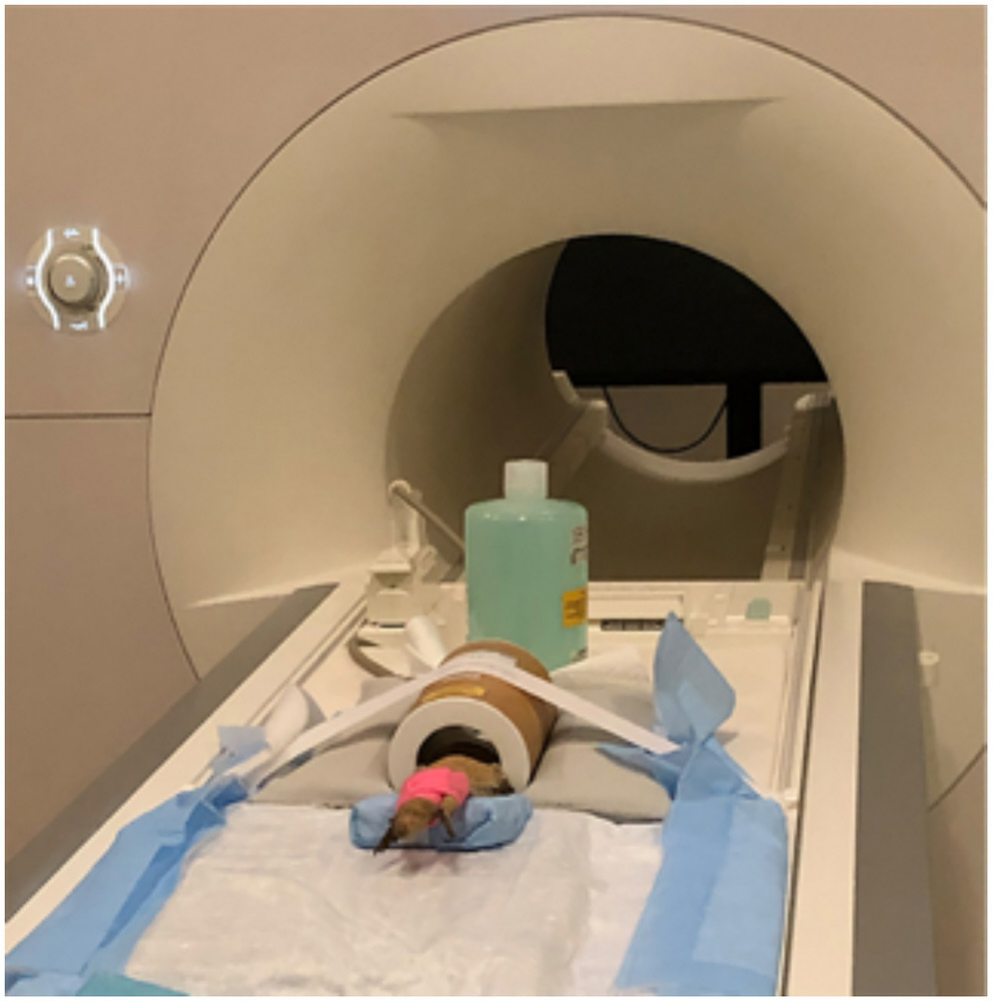
Bearded dragon (BD) positioned in the Rapid Biomed transmit/receive rat coil prior to MRI acquisition. The BD was positioned so the head was approximately halfway in the coil. The lower half of the body was supported on a foam wedge.

All lizards were placed in sternal recumbency within the coil and their heads were covered using a self-adhesive bandage (Vet Wrap) with cotton balls over the eyes to prevent any visual stimulation and provide additional restraint through stimulation of the oculocardiac reflex. The self-adhesive bandage was also placed over the hindlimbs to provide additional restraint and prevent potential movement. The lower half of the lizard was placed on a foam wedge to provide additional body support so that the lizard was not hanging out of the coil. Due to the short acquisition window external heat was not provided and anesthetic monitoring was limited to visual observation of respiration using a camera in the imaging suite. Images were obtained using a 3T MRI (Siemens Prisma, Erlangen, Germany) and a transmit/receive rat coil (Rapid Biomedical, Rimpar, Germany) (Figure 1). A 3D T2-weighted, variable flip angle turbo spin echo (TSE) sequence (3D SPACE) was used to provide a high-resolution 3D scan with 0.23 mm isotropic resolution, TE 285 ms, TR 2.5 s, 59 × 51.6 × 13.8 mm field of view (FOV), turbo factor of 67, with an acquisition time of 15.5 min. In addition, lower resolution and larger field of view T1-weighted (FOV = 96 mm; slice thickness = 1 mm; TR = 15 ms; TE = 3.59 ms; acquisition time of 1.5 min) and T2-weighted (FOV = 100 mm; slice thickness = 0.8 mm; TR = 2.5 s; TE = 148 ms; acquisition time = 6.25 min) acquisitions were obtained for localization of the limited field of view for imaging the brain.

### Averaged Brain Creation

The 3D-SPACE images were converted from the Digital Imaging and Communications in Medicine (DICOM) format to the Neuroimaging Informatics Technology Initiative (NIfTI) format using dcm2niix (https://www.nitrc.org/projects/dcm2nii/) (15). Using the 3D-SPACE images, a binary mask was manually drawn over brain tissue (including CSF) for each lizard using the FSL Eyes function of FSL (v1.3.0; FMRIB Centre, Nufflield Department of Clinical Neuroscience, Oxford, UK) (16).

Brain only masks were manually extracted from the images using ITK Snap (v3.8.0, http://www.itksnap.org/pmwiki/pmwiki.php) (17). An extracted brain from one lizard was selected as the initial target and all brain only extractions were linearly registered to this target using the “flirt” command in FSL (18). These were then averaged together to make an initial linear atlas. All brain only masks were then linearly registered to the new initial linear target using the “flirt” command in FSL and were averaged together to make a final linear atlas of the brain alone.

To compensate for morphological differences among the different lizards, non-linear registration was performed using MATLAB (R2021b) imregdemons (19, 20). The linearly aligned brain only images from each lizard were non-linearly registered to the final linear atlas, with the non-linear warp field saved for each. The warp fields from the atlas to each individual brain were averaged across all lizard brains to find the average warp field. This average deformation field was then applied to the final linear atlas to make an initial non-linear atlas. Finally, all linearly registered lizard brain only images were non-linearly registered to the initial non-linear atlas. Brain only images were warped to the non-linear atlas space along with brain images that include the CSF spaces. All of these individual non-linearly warped images were averaged together to make the final non-linear atlas using the “fslmaths” command in FSL.

### Atlas Creation

To create an atlas with the best possible resolution, we used a non-linear image averaging strategy to create an “idealized” model of a bearded dragon brain. The model represents a significant improvement in the signal-to-noise ratio over the MRIs of individual brains at this high resolution. This atlas can now be used as a standard component of the image registration process for structural MRI analysis of bearded dragons going forward. To further improve the utility of the atlas for localizing regions of interest, we utilized anatomical atlases on tawny dragons (11, 12) the tokay gecko (8), and the garter snake (10) as references, to identify and manually draw nine regions of interest (ROI) on the average brain using FSLEyes. Since there is not a specific bearded dragon brain atlas and the main purpose of this atlas is for clinical use, regions chosen were large, generalized regions that can be easily identified on MRI.

## Results

**Figure 2:**
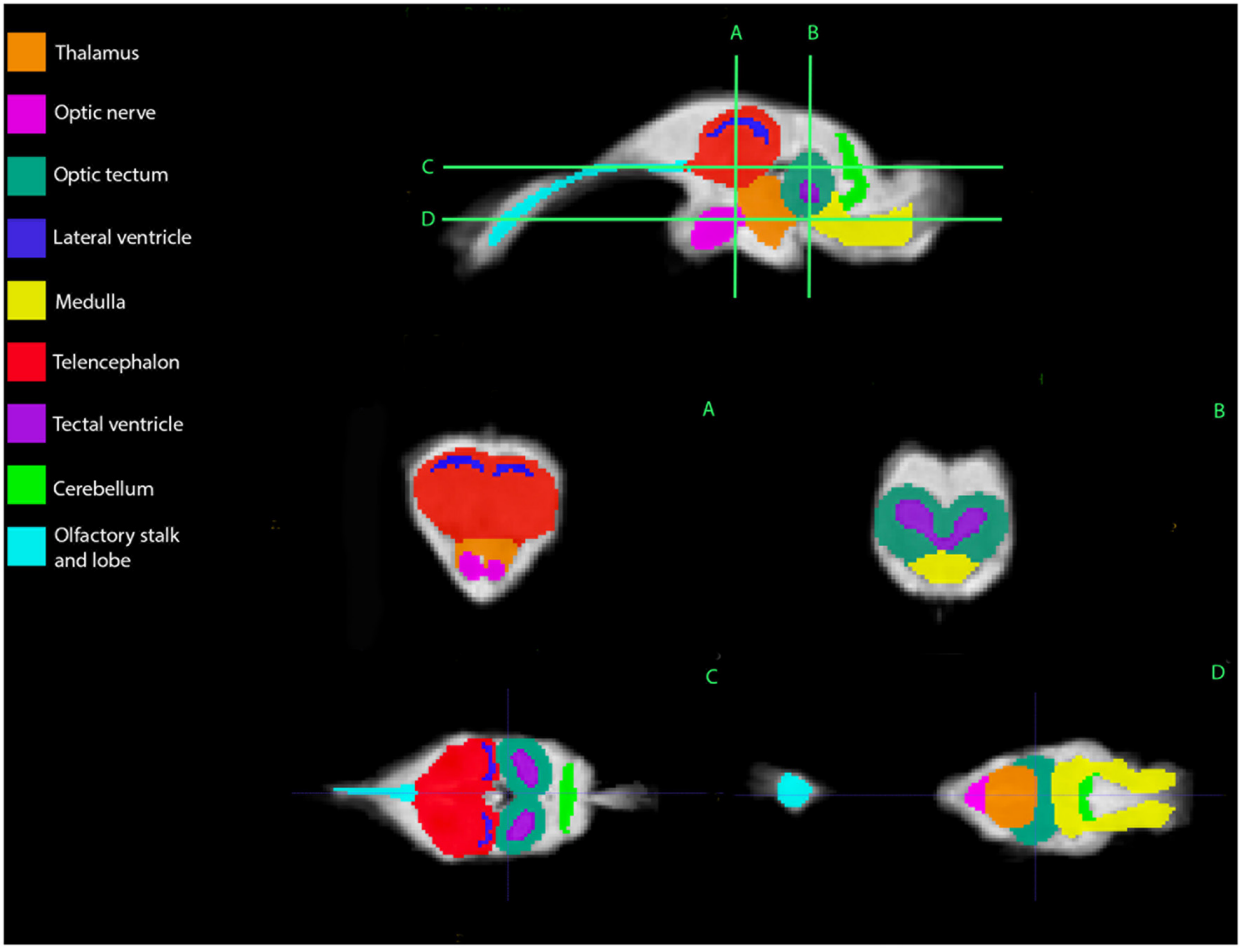
Bearded dragon (BD) regions of interest. A,B—Transverse images at the level of the mid-telencephalon **(A)** and optic tectum **(B)**. C,D—Dorsal images at the level of the telencephalon and optic tectum **(C)** and medulla **(D)**. **(A)** is a parasagittal slice and demonstrates all nine ROI's. **(B)** depicts a transverse slice at the level of the mid-telencephalon and the telencephalon, lateral ventricles, thalamus, and optic nerve can be identified. In **(B)**, the optic tectum, tectal ventricles, and medulla are observed. Doral sequences are displayed in **(C,D)**. The olfactory stalk, telencephalon, lateral ventricles, optic tectum, tectal ventricles, and cerebellum are identified in **(C)**. The olfactory lobe, optic nerve, thalamus, optic tectum, medulla, and cerebellum are visible in **(D)**.

**Figure 3:**
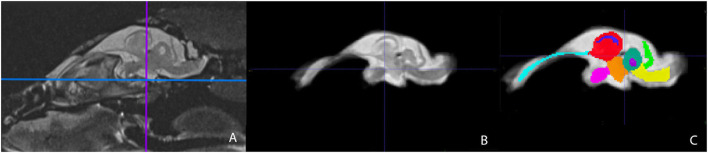
Parasagittal view of the Bearded Dragon brain *in vivo* **(A)**, extracted brain **(B)**, extracted brain with ROIs **(C)**. **(A)** is the acquired MRI sequence of the brain *in vivo* and **(B)** is the extracted warp atlas. **(C)** is the warp atlas with the corresponding ROI's color coded. In **(C)**, all 9 ROI's can be seen.

**Figure 4:**
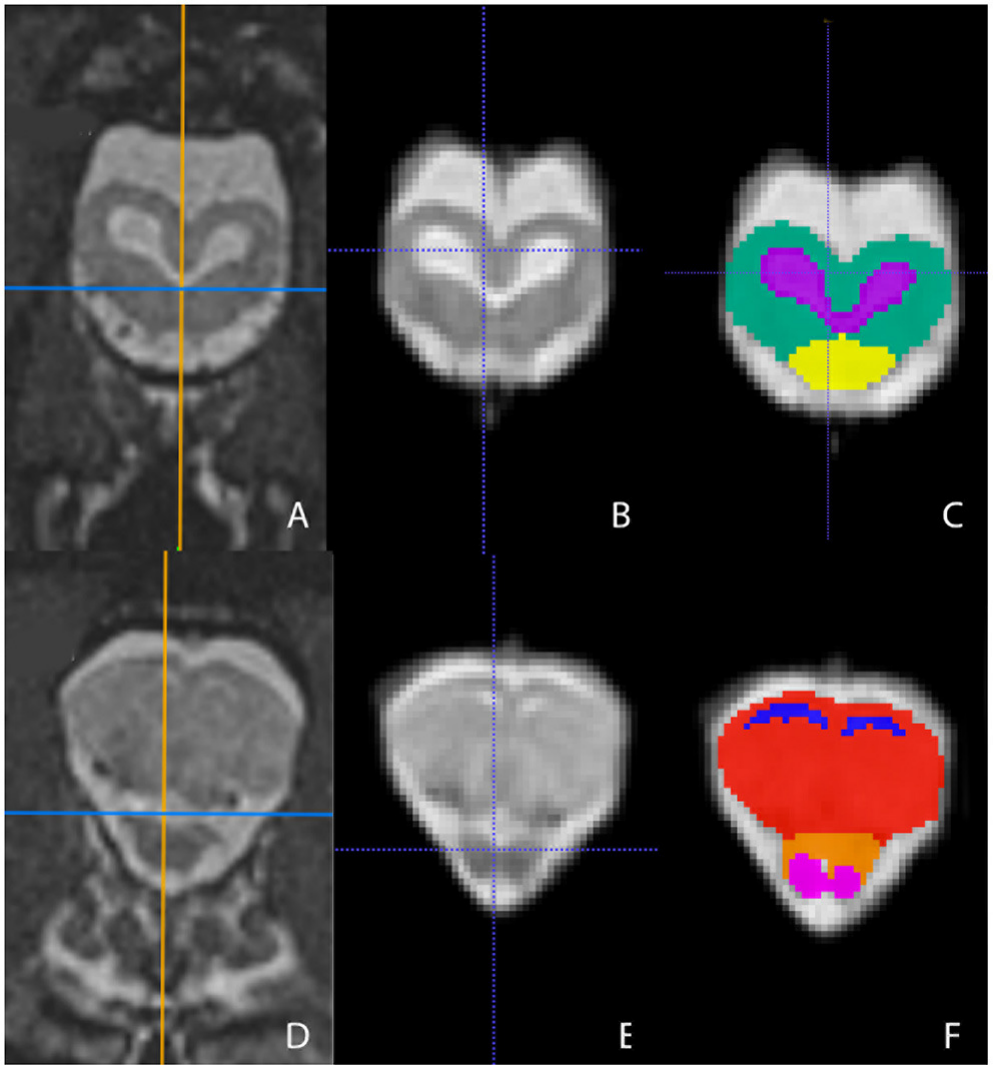
Transverse images at the level of the optic tectum **(A–C)** and mid-telencephalon **(D–F)** demonstrating images *in vivo* **(A,D)**, extracted **(B,E)**, and ROI's **(C,F)**. **(A,D)** are the *in vivo* sequences obtained while **(B,E)** are of the warped atlases with the overlying bone and musculature removed to make a warp atlas. **(C,F)** are the warp atlases with color-coded ROIs. In **(C)**, the optic tectum, tectal ventricles, and the cranial aspect of the medulla are noted. In **(F)**, the lateral ventricles, telencephalon, cranial thalamus, and the optic nerves are seen.

**Figure 5:**
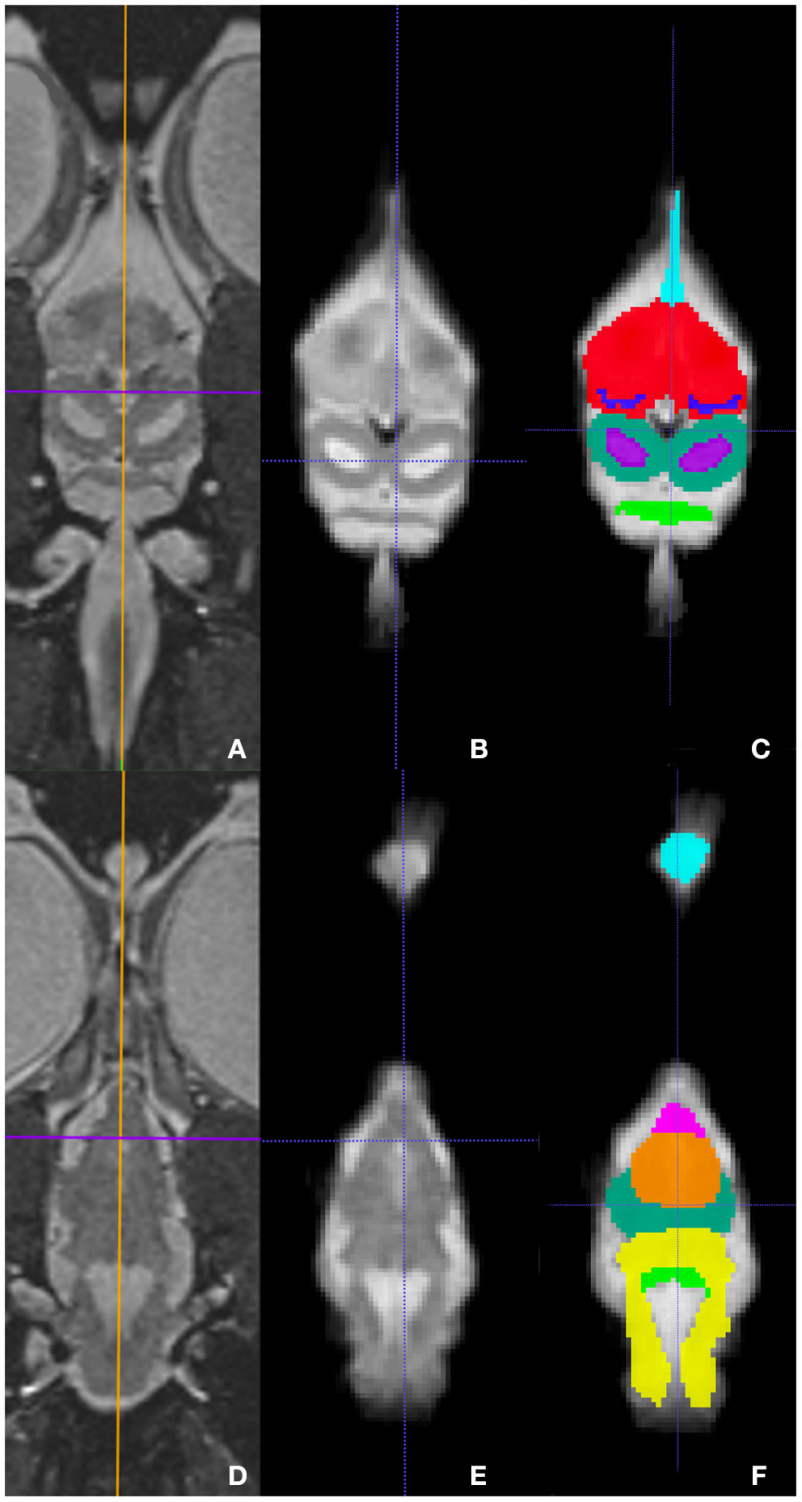
Dorsal images at the level of the telencephalon and optic tectum **(A–C)** and medulla **(D–F)** demonstrating images *in vivo* **(A,D)**, extracted **(B,E)**, and ROI's **(C,F)**. **(A,D)** are the brains *in vivo* and **(B,E)** are the extracted warp atlases. In **(C)** the olfactory stalk, telencephalon, lateral ventricles, optic tectum, tectal ventricles, and cerebellum are identified while in **(F)**, the olfactory lobe, optic nerve, thalamus, optic tectum, medulla, and cerebellum are labeled.

Representative images of the atlas are presented in Figures 2–5. We identified nine anatomic structures in the bearded dragon brain including the thalamus, optic nerve, optic tectum, lateral ventricles, medulla, telencephalon, tectal ventricle, cerebellum, and the olfactory lobe and stalk. The regions of interest labeled are those that are grossly visible on routine MRI and can be easily identified in a clinical setting. Figure 2 displays multiple slices and views, including sagittal, transverse, and dorsal images of the BD brain. Figures 3–5 are representative images of the BD brains *in vivo*, the brains following digital segmentation, and representative regions of interest. In Figure 3, a parasagittal view of the brain is shown. Figure 4 represents transverse images of the brain and Figure 5 shows representative dorsal sequences. It is important to note that images within the manuscript are specifically for exemplary purpose. Images of the entire brain MRIs (as DICOM files) and segmentations in NIFTI format can be accessed in the online Supplementary Material associated with this article.

The total scan time for each lizard was 35 min. Following completion of the MRI, all lizards were clinically hypothermic and apneic. Each lizard was intubated (2.0–2.5 mm uncuffed endotracheal tube) to provide intermittent positive pressure ventilation (IPPV) at 1 respiration/minute with room air until spontaneous ventilation returned; time from intubation to extubation ranged from 15 to 30 min (mean 24.6 min). External heat was provided after MRI acquisition in a similar manner as described above. Heart rate at the time of induction ranged from 70 to 110 bpm (mean 92 bpm) and HR post MRI ranged from 30 to 85 bpm (53 bpm). All lizards recovered uneventfully.

## Discussion

In this study, we developed an MRI-based protocol and brain atlas of the bearded dragon to improve diagnostic capabilities in this species when presenting for neurologic dysfunction. While MRI-based brain atlases of other reptile species exist (10–12, 21), to date none have been produced on the bearded dragon. At this time, the tawny dragon is the only other agamid lizard for which brain atlases have been produced (11, 12), but species differences may still exist.

In the study by Hoops *et al*., imaging was performed on brains that were perfused and fixed *ex vivo* compared this study in which live lizards were imaged. Additionally, the tawny dragon atlas was acquired using an ultra-high-field research scanner (11.74 T) (11, 12), whereas this work was completed using a standard 3T clinical scanner. Thus, providing what can be done in a clinical setting and providing images that match what we would expect in a clinical patient. Given the use of higher field MRI and scanning *ex vivo*, fixed and perfused brains, Hoops *et al*. were able to identify significantly more anatomic structures (over 200), including microscopic areas such as nuclei and fiber tracts. Due to the use of a smaller strength MRI and performing imaging on live patients, we likely cannot detect all possible similarities or differences between these two agamid lizard species (11).

This study focused on imaging of lizard brains *in vivo*, and therefore we were able to include the olfactory lobes and stalks; whereas in the tawny dragon, the olfactory bulbs were unable to be stabilized when removed, and imaging of this structure was not performed. Thus, species comparisons are unable to be made. In the current study, it may have also been easier to identify ventricles as these structures tend to collapse during the fixation process due to CSF no longer being present (11).

All lizards essentially share a basic pattern of brain organization, but morphological, ecological, and behavioral differences between species. One example is that the optic tectum is larger in diurnal lizards compared to those that are nocturnal. Additionally, the size of the cerebellum is related to locomotion type, being smaller in limbless vs. quadrupedal species (11, 22, 23). The cerebellum and optic tectum are well-developed in both the tawny dragon and the bearded dragon as they are quadrupedal, diurnal lizards. Similar to the tawny dragon and other lizard species, the cerebellum in the bearded dragon is also everted (11, 23).

Magnetic resonance (MR) imaging that was initially adopted as a non-invasive imaging technique in people now allows for detailed and rapid analysis of a wide variety of species that have been previously overlooked (21). Additionally, MR is now more sensitive than CT in detecting disease of the central nervous system (CNS) (24). Many facilities with access to clinical MRI have protocols for use in larger companion animals. And while these protocols may be successfully used in dogs and cats, the total duration of MRI acquisition is likely too prolonged to use in most exotic species. This may lead to undesired complications including hypothermia, subclinical respiratory disorders, and possible death (1). The MRI specific protocol developed in this study allowed the acquisition of high quality images with a total scan time of ~35 min.

A limitation to this study is that only a 3D T2-weighted sequence was obtained. When evaluating the brains of clinical patients, commonly used sequences include T1-weighted (pre- and post-contrast), T2-weighted, fluid attenuated inversion recovery (FLAIR), and T2^*^-weighted (susceptibility) (25). For the purpose of this study, we found that this single sequence was adequate to identify anatomic regions within the brain of the bearded dragon at a high spatial resolution and in a tolerable scan time. Additionally, turbo spin echo (TSE) T2 and T1-weighted imaging sequences are considered adequate in providing contrast and spatial resolution for identifying most clinically relevant anatomy (26). While the sequences did provide excellent neuroanatomic correlation and identification of relevant brain morphology, our results do not show the detail found in microscopic histological studies. However, we do see the methods employed as valuable for use in a clinical setting. Another limitation is the neuroanatomic structures were identified using atlases from other reptilian species and not correlated to histological samples from bearded dragons. For the purpose of this atlas, approximate boundaries were identified based on divisions of the brain using the columnar model (11). While boundaries of specific structures may not be completely accurate, the atlas produced in this study still serves its purpose for clinical application.

Another limitation is that while the anesthetic protocol used for the acquisition of images in this study has been shown to have subclinical cardiorespiratory depression (9), the lizards in our study became apneic. However, they were not kept within their preferred optimal temperature zone, and there was likely altered metabolism of the sedative given. Reptiles in general have an increased capability to survive in anoxic conditions with increased anaerobic metabolic capabilities (27, 28). However, the bearded dragons used in our study were healthy individuals and the authors counsel that a debilitated lizard would require additional anesthetic monitoring equipment and general support in a clinical setting to ensure patient safety. Future studies should investigate the utilization of this protocol in clinically ill bearded dragons.

In summary, we were able to successfully perform *in vivo* MRI on the brain of the bearded dragon (*Pogona vitticeps*) in order to produce an anatomic atlas for reference in clinical practice. Additionally, the MRI protocol utilized allowed rapid acquisition of highly detailed images of the bearded dragon brain. Future directions include performing MRI of the bearded dragon brain utilizing a higher strength magnet to further identify more detailed information, in particular, specific nuclei and tracts.

## Data Availability Statement

The original contributions presented in the study are included in the article/Supplementary Material, further inquiries can be directed to the corresponding author/s.

## Ethics Statement

The animal study was reviewed and approved by University of Illinois at Urbana-Champaign Institutional Animal Care and Use Committee.

## Author Contributions

KF and BS were responsible for creation of the atlas and data analysis. All authors were involved in the study design, implementation, data acquisition, and read and approved the final version of the manuscript.

## Conflict of Interest

The authors declare that the research was conducted in the absence of any commercial or financial relationships that could be construed as a potential conflict of interest.

## Publisher's Note

All claims expressed in this article are solely those of the authors and do not necessarily represent those of their affiliated organizations, or those of the publisher, the editors and the reviewers. Any product that may be evaluated in this article, or claim that may be made by its manufacturer, is not guaranteed or endorsed by the publisher.
